# Percutaneous transforaminal endoscopic discectomy combined with acupuncture for lumbar disc herniation: A protocol for systematic review and meta-analysis

**DOI:** 10.1097/MD.0000000000032296

**Published:** 2022-12-23

**Authors:** Shugui Feng, Qiang Yang, Lei Zhao, Guoqiang Wang, Chenyi Yang

**Affiliations:** a Luzhou Traditional Chinese Medicine Hospital Affiliated to Southwest Medical University, Luzhou, Sichuan, China.

**Keywords:** acupuncture, lumbar disc herniation, meta-analysis, PTED, systematic review

## Abstract

Lumbar disc herniation (LDH) is a common orthopedic disease, which is one of the main causes of global chronic pain and motor function defect. For intractable LDH, the surgical treatment of percutaneous transforaminal endoscopic discectomy (PTED) is often used in clinical practice. Although the operation is relatively mature, there may still be some limitations and surgical risks delaying the recovery process of patients. Some studies have found that the combination of acupuncture therapy and PTED can bring more benefits to patients, but these conclusions are still lack of evidence support. Therefore, this study will adopt the method of meta-analysis to estimate the effectiveness and safety of acupuncture combined with PTED in patients with LDH, in order to provide a basis for clinical decision-making.

**Methods:**

This study will conduct a systematic search of the following databases: the Chinese databases China National Knowledge Infrastructure, formerly Chinese Biomedical Database, Wanfang Data, Chongqing VIP Database for Chinese Technical Periodicals, the PubMed, Medline, Embase, Web of Science and The Cochrane library, from their inception to December 1, 2022. In addition, we will search their references to avoid missing other eligible studies. The language will be limited to English and Chinese. RevMan 5.4 will be used to perform the meta-analysis. And GRADE Profiler software will be used to rate the quality of evidence.

**Results:**

The results will be submitted to a peer-reviewed journal.

**Conclusion:**

This study can provide evidence support for acupuncture combined with PTED in the treatment of LDH.

## 1. Introduction

Lumbar disc herniation (LDH) is a common osteosurgical disease. Pain and dysfunction caused by mechanical nerve root compression and chemical stimulation of all types of inflammatory mediators are the most prominent symptoms of LDH. Chronic low back pain is one of the major long-term consequences of LDH, which brings great social and economic pressure.^[1]^ According to the survey, about 9% of the global population is affected by LDH-induced low back pain.^[2]^ Lower back pain is one of the leading causes of reduced productivity and disability in 126 countries worldwide.^[3]^ In current clinical practice, surgery is usually used to treat LDH that is difficult to be resolved by conservative treatment. As a minimally invasive technique, percutaneous transforaminal endoscopic discectomy (PTED) can not only reduce the invasive nature of surgical operations, but also achieve better surgical results as far as possible, reduce surgical injuries and accelerate postoperative rehabilitation of patients. Relevant studies have proved that PTED has good clinical efficacy, so it is gradually promoted in clinical practice.^[4]^ However, there is also evidence-based evidence that there is no significant difference between PTED and the commonly used clinical open microdiscectomy for LDH, which may be related to the potential limitations of PTED.^[5]^

Acupuncture and moxibustion is a representative external treatment of traditional Chinese medicine. There have been quite a number of studies to confirm the effect of acupuncture and moxibustion on analgesia. With the deepening of research and the expansion of clinical indications, some scholars have found that acupuncture and moxibustion can also intervene in the perioperative adjuvant treatment of modern surgery, which has potential advantages in relieving postoperative pain, promoting the recovery of motor function and accelerating rehabilitation.^[6,7]^ Based on the above effects, some clinical workers have combined acupuncture with PTED to treat LDH, showing more prominent postoperative rehabilitation effects.^[8,9]^ This means that patients will have less pain after surgery, a shorter recovery time, and less hospital expenses. However, the combination of acupuncture and PTED in the treatment of LDH still lacks the support of high-quality evidence-based medical evidence, making the effectiveness of this new treatment regimen full of uncertainties. For this reason, this study will conduct a meta-analysis of the current clinical data on the combined treatment of LDH with acupuncture and PTED, and evaluate the effectiveness and safety of this method for the reference of clinical decision makers.

## 2. Methods

This protocol follows the Preferred Reporting Items for Systematic Reviews and Meta-Analysis Protocols (PRISMA-P) guideline.^[10]^ The meta-analysis protocol has been registered on the International Prospective Register of Systematic Review (Prospero CRD 42022373125).

### 2.1. Types of participants

1. Patients who received imaging examination and were diagnosed as LDH according to internationally recognized diagnostic guidelines for LDH will be included.
2. Patients receiving simple conservative treatment for >3 months with no obvious effect and relevant surgical indications will be included.

### 2.2. Types of interventions

The intervention measures in this study were defined as PTED versus Acupuncture. Acupuncture is limited to classic body acupuncture and electroacupuncture, excluding other acupuncture therapies such as scalp acupuncture, ear acupuncture, point injection, point stimulation, etc. PTED generally needs to be carried out under anesthesia, and we have not yet restricted its routine treatment in the perioperative period.

### 2.3. Types of outcomes

#### 2.3.1. Primary outcomes

1. The recent/long-term clinical efficacy.
2. Visual analogue scale score.
3. Japanese Orthopaedic Association low back pain score.

#### 2.3.2. Secondary outcomes

1. Blood biochemical markers, including c-reactive protein and creatine kinase.
2. Nerve conduction function.
3. Reoperation rate.
4. Adverse events.

### 2.4. Types of studies

This study will include all relevant standard RCTs, and quasi-RCTs will be excluded, such as using in clinic order allocation of RCT, case report is not included. Because we believe that the evidence for treatment of non-rigorous RCTS is lower.

### 2.5. Search strategy

We will conduct a systematic search of the following databases: the Chinese databases China National Knowledge Infrastructure, formerly Chinese Biomedical Database, Wanfang Data, Chongqing VIP Database for Chinese Technical Periodicals, the PubMed, Medline, Embase, Web of Science and The Cochrane library, from their inception to December 1, 2022. In addition, we will search their references to avoid missing other eligible studies. The language will be limited to English and Chinese. The literature search will use “(PTED) AND (Acupuncture) AND (LDH)” as the main term combination. Detailed search terms and search strategies are presented in Table 1.

**Table 1:** Search strategy.

Number	Search terms
#1	Acupuncture [Mesh]
#2	Acupuncture [Title/Abstract]
#3	#1 OR #2
#4	Percutaneous transforaminal endoscopic discectomy [Title/Abstract] OR PTED [Title/Abstract]
#5	Lumbar disc herniation [Title/Abstract] OR LDH [Title/Abstract]
#6	randomized controlled trial[Publication Type]
#7	controlled clinical trial[Publication Type]
#8	randomized[Title/Abstract]
#9	randomly[Title/Abstract]
#10	#6 OR #7 OR #8 OR #9
#11	#3 AND #4 AND #5 AND #10

PTED = percutaneous transforaminal endoscopic discectomy.

### 2.6. Literature selection and data extraction

**Figure 1.**
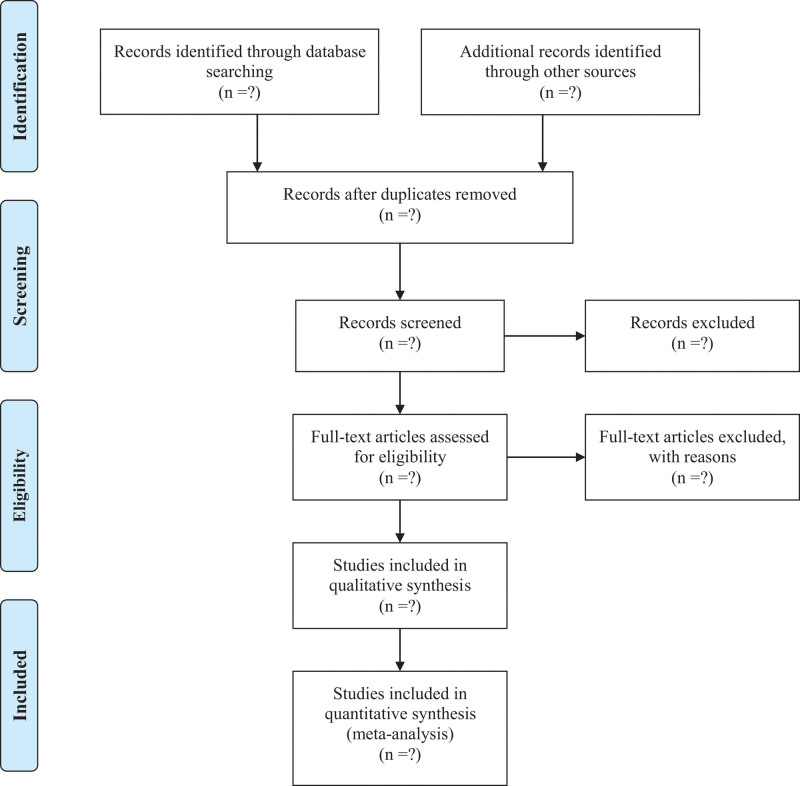
Preferred Reporting Items for Systematic Reviews and Meta-Analysis (PRISMA) flow diagram of study identification and selection.

Two experienced literature researchers (SG Feng and Q Yang) will independently search the database to eliminate duplicate, invalid and any literature that does not meet the inclusion criteria. The final RCTs are recorded with the preset data extraction tool. The whole process of literature screening is shown in Figure 1. The data to be extracted includes:

1. Research characteristics: title, journal, first author name, random method;
2. Participants information: sample size, gender, mean age, race, diagnostic criteria, disease course;
3. Intervention information: type of therapy, clinical dosage, course of treatment;
4. Clinical outcomes: primary and secondary outcomes.

### 2.7. Risk of bias assessment

Use the standards recommended by the Cochrane Collaboration to evaluate the bias risk of the final included literature. Two other independent researchers (L Zhao and GQ Wang) carried out the above process, and the evaluation contents include: randomization process, deviations from the intended interventions, missing outcome data, measurement of the outcome, selection of the reported outcome. In case of any discrepancy, it shall be settled through negotiation or submitted to the corresponding author for review and ruling.

## 3. Data analysis

As this study only includes strict RCTs, it may cause a certain risk of bias. Therefore, the data analysis results of this study should be carefully evaluated.

### 3.1. Selection of effect measure

In this study, continuous outcome variables including visual analogue scale score, Japanese Orthopaedic Association low back pain score, blood marker level and nerve conduction function will use the standardized mean difference as the effect measure. dichotomous variables including clinical efficacy, reoperation rate and adverse events will use odds ratio to evaluate the effect measure. All effect amount will be expressed in a 95% confidence interval.

### 3.2. Statistical heterogeneity

Check and count the heterogeneity by inspecting the Forest plot, and assess the size of the heterogeneity by *I*^2^. Heterogeneity will not be considered when *I*^2^ value is <50%. On the contrary, when *I*^2^ value is >50%, we believe that there is a large heterogeneity, and we will further analyze the source of heterogeneity.

### 3.3. Subgroup and sensitivity analyses

When the heterogeneity is too large, we will explore the possible sources of significant inconsistencies or heterogeneity through meta regression analysis and grouping. The confounding factors that need to be analyzed include but are not limited to age, anesthetic dosage, operation time, blood loss, acupuncture intervention point of time and follow-up time.

### 3.4. Meta-analysis

When the heterogeneity is relatively small, we will further conduct a meta-analysis of the outcomes of all RCTs one by one. When multiple outcomes were available from a single study, the value was used which was thought to be best correlated to that time interval. A random effect model will be used for all analyses based upon the DerSimonian and Laird approach.^[11]^ RevMan 5.4 (The Nordic Cochrane Center, The Cochrane Collaboration, Denmark) will be used to perform the meta-analysis.

### 3.5. Publication bias

If >10 studies are finally included, we will draw a funnel chart to assess publication bias. The Egger test was then used to assess the asymmetry of the funnel plot.

### 3.6. Grading the quality of evidence

Based on the 5 parts (limitations of design, inconsistency of results, indirectness, imprecision, and other factors), we will evaluate the evidence quality of all outcomes. The quality of evidence is divided into 4 levels: very low, low, model, and high. GRADE profiler software will be used for the above evidence quality grading.^[12]^

## 4. Discussion

This study was to verify the acupuncture combined with PTED efficacy and safety of the treatment of LDH, to help clinical decision making. PTED is the mainstream method for treating LDH in recent years. There is evidence that perioperative management of acupuncture intervention can promote postoperative recovery of patients. However, there is no meta-analysis evidence for acupuncture combined with PTED. Therefore, this study hopes to explore this, in order to expand the application scope of acupuncture as an auxiliary alternative therapy, and provide a new scheme for the postoperative management of clinical surgery patients.

## Author contributions

SG Feng was responsible for this study. SG Feng and CY Yang conceived and designed the study. SG Feng, L Zhao, Q Yang and GQ Wang participated in drafting the protocol and preparing the manuscript. All authors read and approved the final manuscript.

**Conceptualization:** Shugui Feng, Lei Zhao, Guoqiang Wang, Chenyi Yang.

**Data curation:** Shugui Feng.

**Formal analysis:** Qiang Yang, Lei Zhao.

**Methodology:** Shugui Feng, Qiang Yang, Lei Zhao, Guoqiang Wang.

**Software:** Guoqiang Wang.

**Writing – original draft:** Shugui Feng, Qiang Yang, Lei Zhao, Chenyi Yang.

**Writing – review & editing:** Shugui Feng, Guoqiang Wang, Chenyi Yang.
